# The use of PET-MRI in the follow-up after radiofrequency- and microwave ablation of colorectal liver metastases

**DOI:** 10.1186/1471-2342-14-27

**Published:** 2014-08-08

**Authors:** Karin Nielsen, Hester J Scheffer, Indra C Pieters, Aukje AJM van Tilborg, Jan-Hein TM van Waesberghe, Daniela E Oprea-Lager, Martijn R Meijerink, Geert Kazemier, Otto S Hoekstra, Hermien WH Schreurs, Colin Sietses, Sybren Meijer, Emile FI Comans, Petrousjka MP van den Tol

**Affiliations:** 1 Department of Surgery, VU University Medical Center, De Boelelaan 1117, 1081 HV Amsterdam, The Netherlands; 2 Radiology & Nuclear Medicine, VU University Medical Center, De Boelelaan 1117, 1081 HV Amsterdam, The Netherlands; 3 Department of Surgery, Medical Center Alkmaar, Postbus 501, 1800 AM Alkmaar, The Netherlands; 4 Department of Surgery, Gelderse Vallei, Willy Brandtlaan 10, 6716 RP Ede, The Netherlands

**Keywords:** Radiofrequency ablation, Liver neoplasms/secondary, Neoplasm recurrence, Local, Liver neoplasms/surgery, FDG-PET, PET-MRI, Magnetic resonance imaging/methods, Microwave ablation

## Abstract

**Background:**

Thermal ablation of colorectal liver metastases (CRLM) may result in local progression, which generally appear within a year of treatment. As the timely diagnosis of this progression allows potentially curative local treatment, an optimal follow-up imaging strategy is essential. PET-MRI is a one potential imaging modality, combining the advantages of PET and MRI. The aim of this study is evaluate fluorine-18 deoxyglucose positron emission tomography (FDG) PET-MRI as a modality for detection of local tumor progression during the first year following thermal ablation, as compared to the current standard, FDG PET-CT. The ability of FDG PET-MRI to detect new intrahepatic lesions, and the extent to which FDG PET-MRI alters clinical management, inter-observer variability and patient preference will also be included as secondary outcomes.

**Methods/Design:**

Twenty patients undergoing treatment with radiofrequency or microwave ablation for (recurrent) CRLM will be included in this prospective trial. During the first year of follow-up, patients will be scanned at the VU University Medical Center at 3-monthly intervals using a 4-phase liver CT, FDG PET-CT and FDG PET-MRI. Patients treated with chemotherapy <6 weeks prior to scanning or with a contra-indication for MRI will be excluded. MRI will be performed using both whole body imaging (mDixon) and dedicated liver sequences, including diffusion-weighted imaging, T1 in-phase and opposed-phase, T2 and dynamic contrast-enhanced imaging. The results of all modalities will be scored by 4 individual reviewers and inter-observer agreement will be determined. The reference standard will be histology or clinical follow-up. A questionnaire regarding patients’ experience with both modalities will also be completed at the end of the follow-up year.

**Discussion:**

Improved treatment options for local site recurrences following CRLM ablation mean that accurate post-ablation staging is becoming increasingly important. The combination of the sensitivity of MRI as a detection method for small intrahepatic lesions with the ability of FDG PET to visualize enhanced metabolism at the ablation site suggests that FDG PET-MRI could potentially improve the accuracy of (early) detection of progressive disease, and thus allow swifter and more effective decision-making regarding appropriate treatment.

**Trial registration:**

Trial registration number: NCT01895673

## Background

Thermal ablation techniques, including radiofrequency ablation (RFA) and microwave ablation (MWA), are now well-established therapeutic options for the localized treatment of unresectable colorectal liver metastases (CRLM) [1]. Although potentially curative in selected cases, these techniques carry a risk of incomplete ablation and can show local tumor progression (LTP) rates of up to 30%, of which >95% are diagnosed within one year of ablation [2-4]. Complete tumour clearance, and therefore potential cure, can still be achieved in patients without extensive intra or extrahepatic metastases through re-treatment of a LTP [1,5].

Imaging plays a key role in diagnosing progressive disease following hepatic RFA and MWA, with post-ablative lesions generally monitored using contrast-enhanced computed tomography (ceCT) and magnetic resonance imaging (MRI). Without proof of lesion growth from consecutive scans, an important weakness of these modalities is the inability to distinguish between reactive tissue surrounding the ablated lesion (post-ablation effects) and viable tumour [6]. Diffusion-weighed (DW) MRI outperforms ceCT in the initial detection of small hepatic metastases (<1 cm) and is also superior to ceCT in the detection of CRLM after treatment with systemic chemotherapy without radiation load [7,8].

Due to the visualization of increased glucose metabolism in tumour cells, fluorine-18 deoxyglucose positron emission tomography (FDG-PET) is a useful tool in the assessment of treatment response following RFA [6,9]. Ablated lesions that show focal FDG uptake in or within 1 cm of the ablated area are considered clinically suspect for LTP [6,10]. However, as FDG-PET lacks an anatomical reference it is less accurate in determining the exact location of viable tumour tissue. A solution to this problem was found by combining FDG-PET and CT images to provide fused functional and morphological data [11], and PET-CT has since been shown to be superior to ceCT in the identification of LTP [6,9]. Integrated PET-MRI is a new imaging modality, combining the advantages of FDG-PET with the ability of MRI to detect small liver tumours without CT radiation exposure. Recent results show that detection sensitivity for hepatic metastases, through post hoc fusion of FDG-PET images and 1.5 Tesla contrast-enhanced (Gadolinium) MRI obtained from two different scanners, is significantly higher than for PET-CT (93% and 76% respectively, p = 0.02) [12]. PET-MRI appears to offer higher lesion conspicuity and diagnostic confidence compared to PET-CT [13], and this additional information can influence clinical management of cancer patients The combined advantages of detection of smaller hepatic tumours with a higher sensitivity and detection of focal FDG uptake suggestive for LTP indicates that PET-MRI could provide complementary information and facilitate improved clinical decision making [14].

To the best of our knowledge, the effectiveness of integrated PET-MRI in RFA or MWA follow-up has not been investigated. The primary aim of this study is therefore to determine the accuracy of 18 F-FDG PET-MRI in LTP detection. Secondary aims will be to investigate whether PET-MRI is more accurate than PET-CT and ceCT in the detection of new intra-hepatic lesions, and to evaluate inter-observer variability, the impact of PET-MRI on future treatment and patients’ views on both modalities. We hypothesize that PET-MRI will be at least equivalent to PET-CT in the diagnosis of LTP, but superior to PET-CT in the early detection of new intrahepatic lesions and therefore able to positively influence decision making in patients with RFA/MWA-treated CRLM.

## Methods/Design

### Design

This prospective study has been approved by the Medical Ethical Review Board of the VU University Medical Center. The study is investigator-sponsored, independent of industry and is registered at clinicaltrials.gov under number NCT01895673.

### Primary and secondary objectives

We will assess the effectiveness of FDG PET-MRI in the follow-up of thermal ablation (RFA/MWA) of CRLM. Our primary aim will be to assess the accuracy of FDG PET-MRI in the diagnosis of local progression in the first year after RFA/MWA of CRLM. Therefore, all FDG PET-MRI images will be compared to FDG PET-CT and 4-phase liver CT images, which are the current standard of care. Results of different imaging sets will be compared on a per-patient and a per-lesion base. These results will then be compared to a reference standard: either histological outcome (if available) or clinical follow-up.

Secondary outcomes will include the accuracy of diagnosis of new intrahepatic disease after RFA/MWA. Since only a single 18 F-FDG per combined PET-MRI and PET-CT visit will be applied, we will have the opportunity to investigate whether dual-time point PET can increase the specificity of the 18 F-FDG signal and increase diagnostic accuracy [15]. The reproducibly and robustness of inter-observer variation will also be determined. Clinical relevance will be studied by recruiting a panel consisting of a radiologist, interventional radiologist, nuclear medicine physician, oncologist, oncological surgeon and a radiation oncologist to retrospectively review PET-MRI images and agree on the preferred treatment. Choice of treatment based on PET-MRI will then be compared to the treatment that was actually received based on PET-CT and 4-phase liver CT. Finally, patients’ preferences will be assessed at one year follow-up using a specifically designed questionnaire that compares both modalities (Table 1).

**Table 1:** Questionnaire comparing PET-CT and PET-MRI (completed following fourth PET-MRI)

**Questions about the PET-MRI**							
It was comfortable	0	1	2	3	4	5	n/a
I felt scared	0	1	2	3	4	5	n/a
It took too long	0	1	2	3	4	5	n/a
The noise bothered me	0	1	2	3	4	5	n/a
I was reluctant to undergo the scan	0	1	2	3	4	5	n/a
It went better than expected	0	1	2	3	4	5	n/a
The PET-MRI contrast agents caused discomfort	0	1	2	3	4	5	n/a
**Questions about the PET-CT**							
It was comfortable	0	1	2	3	4	5	n/a
I felt scared	0	1	2	3	4	5	n/a
It took too long	0	1	2	3	4	5	n/a
The noise bothered me	0	1	2	3	4	5	n/a
I was reluctant to undergo the scan	0	1	2	3	4	5	n/a
It went better than expected	0	1	2	3	4	5	n/a
The PET-CT contrast agents caused discomfort	0	1	2	3	4	5	n/a
**Comparing PET-CT to PET-MRI**							
The PET-MRI was less of a burden than the PET-CT	0	1	2	3	4	5	n/a
It didn’t matter that the PET-MRI took longer than the PET-CT	0	1	2	3	4	5	n/a
If the results are equally good, I prefer the PET-MRI over the PET-CT	0	1	2	3	4	5	n/a
If the results of the PET-MRI are *better* than the PET/CT, I would prefer the PET-MRI over the PET-CT	0	1	2	3	4	5	n/a
**Comments**							

0: Don’t know. 1: Strongly disagree. 2: Disagree. 3: Neither agree nor disagree. 4: Agree. 5: Strongly agree. n/a: not applicable.

### Patients

Written informed consent will be obtained from all participants. Patients scheduled to undergo either RFA/MWA of CRLM, solely or in combination with resection, or RFA/MWA for LTP after previous thermal ablation of CRLM, will be recruited at a referral university hospital (VU University Medical Center, Amsterdam, the Netherlands) and a large community teaching hospital (Gelderse Vallei, Ede, the Netherlands). Assessment prior to ablation consists of a full-body 18 F-FDG PET-CT and 4-phase liver CT or MRI of the liver. Increased FDG uptake of the CRLM on the pre-operative PET scan is a requirement. All patients will be discussed by a multidisciplinary liver board prior to treatment and suitability for RFA or MWA will be at their discretion. Treatment will depend, amongst others things, on the number, location and size of lesions. An open approach using intra-operative ultrasound is preferred as an initial treatment, and a CT-guided percutaneous approach will be used for treatment of recurrences. Technical success, or was the tumor ablated according to protocol, was required before inclusion [16]. Our protocol defines a complete ablation as the entire size of the tumor plus a 1 cm tumor free margin as seen on IOUS or CT. This area was calculated during the planning phase of the procedure.

Exclusion criteria are chemotherapy within 6 weeks prior to the first scan, allergies to contrast media and general contraindications for MRI. Patients with an eGFR <60 will be admitted for additional hydration before and after the scans. If chemotherapy is initiated or if the ablated lesion is resected during the first year of follow-up, participation in the study will be aborted.

### Study procedure

**Figure 1:**
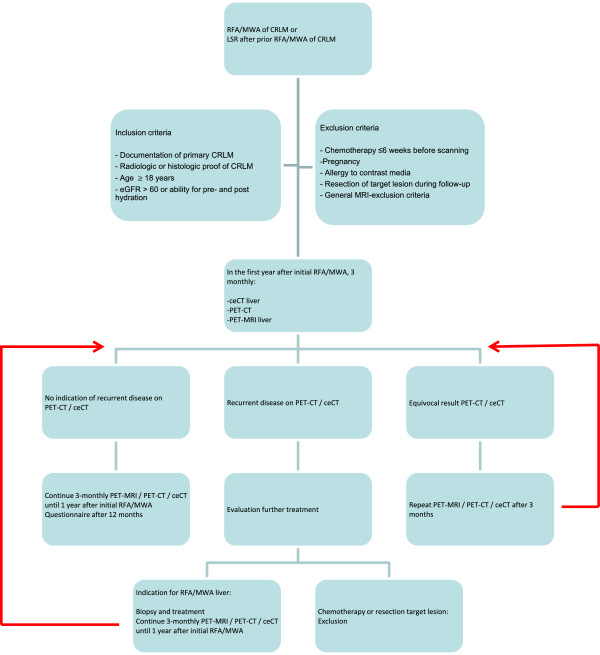
Flow diagram of study procedure.

The standard follow-up protocol during the first year after RFA/MWA of CRLM in the VU University Medical Center consists of three-monthly whole-body FDG PET-CT and 4-phase liver CT, in combination with measurement of serum carcinoembryonic antigen (only when elevated pre-operatively). For the purposes of this study, liver FDG PET-MRI will be added to the routine procedure, using a 3.0 Tesla Ingenuity TF Philips scanner (Philips Medical Systems, Cleveland, OH). This hybrid PET-MRI system is characterized by a sequential acquisition mode and has separate scanners with a turntable patient handling system facilitating patient motion between the MRI and the PET gantries that are located at a 3 meter distance from each other.At the end of one year follow-up, patients will be asked to complete a questionnaire evaluating their experience with the PET-MRI compared to the PET-CT. The study procedure is illustrated in Figure 1.

### Imaging

A summary of imaging steps is provided in Table 2. PET imaging will be conducted according to EANM guidelines [17]. 18 F-FDG will be administered while the patient is supine on the PET-MRI table using MR compatible devices. The MRI will be performed during the period in which FDG distributes throughout the body, and PET will commence approximately 60 minutes after FDG injection. The PET of the PET-CT will follow 20 minutes (±5) after completion of the PET-MRI, followed by a whole body low-dose CT for attenuation and image-fusion and a diagnostic 4-phase liver CT. This scanning sequence has been chosen to minimize the burden for the patient, to optimize reliability of the dedicated liver MRI sequences and to minimize the time between both PET scans.

**Table 2:** Imaging protocol and timeline of PET-MRI and PET-CT (T = approximate time in minutes)

T = 0	Injection FDG	
T = 5	Start MRI	Axial T2-weighted imaging
		T1 in- and opposed phase GRE
		Diffusion-weighted imaging
		Fat-suppressed T1-weighted eThrive (contrast enhanced)
		- pre-contrast
		- arterial phase (bolus tracking)
		- portovenous phase (70 sec delay)
		- late venous phase (200 sec delay)
		Attenuation sequence (skullbase – midthigh)
		Whole body T1-weighted mDixon (skullbase – midthigh)
T = 60		PET skullbase – midthigh
T = 75	**End PET-MRI**	**Transfer to PET-CT**
T = 95	Start PET-CT	PET skullbase – midthigh
		low dose CT
		4-phase CT liver
		- pre-contrast
		- arterial phase (bolus tracking)
		- portovenous phase (70 sec delay)
		- late venous phase (240 sec delay)
T = 145	End PET-CT	

#### MRI

A 16-channel Sense Torso XL coil is connected and a low-resolution non-diagnostic MR attenuation sequence (atMRI) (skull base to mid-thigh) is run, followed by a whole body T1-weighted mDixon sequence, scanned in the axial plane to allow for anatomical matching of FDG images [18]. These images are routinely reconstructed in the coronal plane. Dedicated axial liver imaging is performed: axial T2-weighted TSE (TR/TE 850/80 msec); T1 in- and opposed phase GRE (TR/TE 180/2.3/1.15 msec) and diffusion-weighted imaging (TR/TE 2407/53 msec) using b-values (0, 50 and 800 sec/mm^2^). This is followed by a fat-suppressed T1-weighted GRE using IV Gadolinium-chelate at 0.1 mmol/kg (Dotarem, 0.5 mmol/ml; Guerbet, Gorichem, The Netherlands) in 4 separate phases: pre-contrast, arterial phase (determined by a bolus tracking method to ensure optimal arterial enhancement, which includes full enhancement of the hepatic artery and only partial enhancement of the portal vein [19]), as well as 70 and 200 msec post-injection. Total imaging time for the dedicated liver protocol is approximately 45 minutes.

#### 18F-FDG PET (PET-MRI)

After MRI, the PET-MRI table is turned and the patient is placed in the PET gantry. The PET features an 18 cm axial field of view, 9 cm overlap between bed positions and a 5.5 mm reconstructed isotropic spatial resolution. The scan trajectory covers the skull base to mid-thigh, taking 2 min/bed position. Reconstructed images have an image matrix size of 144X144, voxel size of 4X4 mm and slice thickness of 5 mm. Data are reconstructed by means of a Blob-OS-TF algorithm. Fusion images are created with the PET sequence and atMRI, mDixon axial and coronal whole body and axial T2 liver.

#### 18F-FDG PET (PET-CT)

The Philips Gemini TF PET-CT system (Philips Medical Systems, Cleveland, Ohio, USA) is comparable to the PET unit of the PET-MRI. Scanning is started with a whole body PET (skull base to mid-thigh), with data being reconstructed by means of a raw action ordered subset expectation maximization algorithm using default reconstruction parameters. Time of flight (TF) information is used during reconstruction. Reconstructed images have an image matrix size of 144X144, a voxel size of 4X4 mm and a slice thickness of 5 mm. The acquisition time per bed position is adjusted for the time interval between the start of PET-CT and FDG injection and will usually be 2–3 min/bed position.

#### Low-dose CT

This is followed by a low-dose CT for attenuation and image fusion and is collected using a beam current of 30–50 mAs at 100 keV. The low-dose CT is reconstructed using an image matrix of 512X512, resulting in voxel sizes of 1.17X1.17 mm and a slice thickness of 5 mm.

#### 4-phase liver CT

A diagnostic 4-phase CT of the liver using 100 ml Xenetix300 (Guerbet, Villepinte, France) is subsequently performed, using a beam current of 175-220mAs per slice. First, a precontrast scan is run, followed by the arterial phase using a bolus-tracking method and a 80 keV scan to ensure optimal arterial enhancement [20]. During the venous phase, 70 sec post-injection, the entire abdomen is scanned, followed by a scan of the liver in the late venous phase (240 sec post-injection), both at 120 keV. The images are reconstructed using a matrix of 512X512 and a slice thickness of 3–5 mm. All images are sent to a digital picture archive and communication system (PACS; Sectra AB, Linköping, Sweden).

### Image analysis

Only PET-CT and 4-phase liver CT images will be analysed and used in the context of routine patient care during clinical follow-up. The PET-MRI images will not be part of the diagnostic and follow-up process.

For imaging analysis, blinded data sets of all scans will be viewed both separately and independently by two designated, experienced radiologists and two nuclear medicine physicians. The reviewers will be blinded to the results of the other imaging modalities and to final patient outcome. A time-interval of at least four weeks will be introduced between the first and second review. Progressive disease will be scored on a per-patient and per-lesion basis, and results of all imaging modalities will be compared. The criteria for progression on different modalities are defined in Table 3. Each lesion on each modality will be scored on a separate form and results will be scored from 1–5 (Table 4) and size and location of the lesions will be reported. A LTP is defined as a lesion located in or within 1 cm of the ablation zone; a new intrahepatic lesion is defined as a lesion located more than 1 cm distant from the ablation zone [6]. On MRI, a suspicious lesions has to meet the definition of LTP in at least two different sequences before it is considered positive for progression.

**Table 3:** Test positivity criteria for progressive disease on different imaging modalities; ≤1 cm of the ablation zone (LTP) / >1 cm of the ablation zone (new intrahepatic recurrence)

ceCT		Newly detectable hypodense lesion
MRI	T1	New focal hypo-intense lesion
	T2	New focal hyper-intense lesion
	Contrast enhanced	Irregular peripheral enhancement pattern of a circumscribed lesion
	Diffusion weighed	Lesion with high signal intensity on b800
FDG PET		Lesion with clearly increased focal uptake as compared to liver background

**Table 4:** Study form for reviewers’ results (per lesion base)

**Study number:**		**Reviewer number:**
**Score**	**Definition**	**Explanation**
1	Normal	Confident that no tumor recurrence is present in the ablation zone/confident that no new intrahepatic lesions are present
2	Probably benign	The appearance of the ablated lesion is compatible with post-ablational inflammation or rim-like characteristics/the new lesions diagnosed do not appear malignant
3	Equivocal	There is doubt whether the enhanced FDG, CT- and/or MRI features indicate tumor progression or inflammation/there is doubt whether the new lesions diagnosed are benign or malignant
4	Probably malignant	Confident of local progression in the ablation zone/confident of new intrahepatic metastases
		Size or estimated size:
		Location:
		Intensity FDG vs normal liver; slight/moderate/intense
5	Impaired quality	Quality of the images precludes adequate diagnosis
Comments:		

### Recurrent disease and repeated treatment

If recurrent disease is suspected on either 4-phase CT of the liver or on PET-CT, the patient will be evaluated for re-treatment by our multidisciplinary liver board. If either resection of a LTP or chemotherapy is indicated, the patient will be excluded from further participation in the study. If patients are eligible for repeated RFA/MWA treatment of the LTP, study participation will be continued. Patients with new intrahepatic lesions that are eligible for local treatment (surgery or ablation) will remain in the study, as long as the initially ablated area remains unaffected (Figure 1). Prior to re-treatment with RFA/MWA, several biopsies will be taken from the suspected area for pathological confirmation. To minimize the chance of ‘track seeding’, tract ablation will be performed after the procedure. Extra-hepatic disease will be treated as deemed suitable. If there is any doubt as to whether an imaging feature indicates recurrent disease, the scan will be repeated after 3 months as a confirmation.

### Sample size calculation

Sample size estimates are based on the incidence of local site recurrences and new intrahepatic metastases. Previous studies have shown that 25-40% of the patients treated with RFA/MWA develop LTP, of which >95% occur within the first year [4]. In addition, 30-40% of the patients will develop new intrahepatic metastases [2,21]. At least 5 LTP and 5 new intrahepatic lesions are necessary for a meaningful comparison between PET-CT and PET-MRI in this observational study. Using the binominal distribution (with *p* = 0.35 and n = 20), the chance of finding 5 LTP and the chance of finding 5 new intrahepatic lesions in our population are both 88%. This suggests that the study has a satisfactory chance of success with a sample size of 20 patients. Patients excluded during the study period will be replaced by new participants, so that 20 patients will have completed one year follow-up at study completion.

### Statistical analysis

We will determine the accuracy of PET-MRI for diagnosing recurrent disease compared to each imaging method (MRI, CT and PET-CT) and compared to pathology or clinical follow-up. Continuous variables will be summarized with standard descriptive statistics including means, standard deviations, medians and ranges when indicated. Categorical variables will be summarized with frequencies. McNemar’s test is used to determine agreement between PET-MRI and other modalities. The inter-observer variability will be quantified with Cohen’s Kappa and proportions of specific agreement. P-values less than 0.05 will be considered statistically significant. Statistical analysis will be performed using SPSS version 20.0 (SPSS Inc, Chicago).

## Discussion

Accurate staging is the foundation of prognostic outcomes in oncology and has an immediate effect on patient care in every phase of disease. Although the quality of imaging has improved considerably over the past decades, the search for optimized modalities for specific indications continues. Although the recent introduction of PET-MRI combines the major advantages of both techniques, the value of PET-MRI in staging for the oncological patient still has to be proven. Ours will be the first study to assess the value of PET-MRI in the follow-up of thermal ablation of CRLM.

Early diagnosis of small volume recurrences is the primary goal of intense surveillance strategies following local hepatic tumor ablation. Treatment of these LTP and of new intra-hepatic lesions may allow complete tumor eradication and therefore provide potential cure in patients treated for CRLM [5]. To detect recurrent disease at an early stage, we advocate a local surveillance protocol with intensive follow-up over the 12-month period in which most recurrences occur [4]. Although numerous studies have attempted to define an optimal imaging algorithm after local treatment of liver metastases, the frequency, duration and survival benefit of surveillance have not yet been formally proven and general consensus is lacking [22,23]. To the best of our knowledge, no prospective studies comparing follow-up schemes after RFA or MWA have yet been published.

PET-CT has proven value in the imaging-based diagnosis of recurrent disease following RFA/MWA of liver malignancies, and repeat treatment is often initiated solely based on this imaging modality.

The importance of histological proof of malignancy of a recurrence detected by imaging has long been a subject of debate within the thermal ablation field. We will therefore take multiple biopsies of the suspected area, taking into account the possibility of sampling necrotic tissue rather than active tumor tissue (sampling error). A sampling error of this type may influence our calculation of the specificity of PET-MRI, because false-positive results cannot be completely ruled out. The reference standard (in addition to pathology) for imaging accuracy will be the presence or absence of recurrent disease at clinical follow-up in the first year.

The period between FDG injection and acquisition of the PET-CT in this study is 30 minutes longer than the standard protocol, which might increase our ability to detect intrahepatic malignancies. Background activity in the normal liver parenchyma due to high glucose metabolism and abundant expression of Glut-1 and Hexokinase II can often impede detection of liver lesions. Following a rationale that FDG accumulates over time in malignant cells as compared to healthy or inflamed hepatocytes, several authors have proposed that PET-CT imaging may improve lesion detectability over time through an improvement in the tumor-to-background ratio [24,25]. It is known that FDG uptake by a tumor may not plateau before 90–120 minutes and peak levels may persist for up to 4 hours after injection [26]. Using target-liver ratios, we will investigate whether the 30 minutes delay between both PET-scans contribute to an improved differentiation of post-ablation inflammation and residual tumor [15]. The study has not been designed to provide semi-quantitative values for FDG uptake as predictors of therapeutic response, generally expressed as the standard uptake volume (SUV). However, the SUV can vary considerably and the time lapse between FDG injection and acquisition is a major contributor to this variation [27].

Follow-up is only worthwhile when patients comply to a physician’s recommendations, and compliance with medical advice has been a challenge since first described by Hippocrates [28]. Numerous factors determine whether a person’s behaviour coincides with medical advice, such as the duration of therapy, the complexity of the regimen and psychosocial factors [29]. Adherence to follow-up can also be affected by a patients’ negative experience of examinations such as discomfort and pain [30,31]. These patient preferences may contribute to final decision making on follow-up imaging, especially when modalities are known to be equally effective. We formulated the hypothesis that PET-MRI provides a less favourable experience when compared to PET-CT, as the MRI examination may elicit anxiety due to the need to remain immobile and alone while enclosed in the MRI tunnel for a prolonged period of time, in addition to the noise associated with the examination [32].

In this study, we solely focus on the ability of PET-MRI to identify intrahepatic recurrences, since most recurrences after local treatment of the liver reoccur in the liver [1,2]. However, a large group of patients will eventually develop extrahepatic metastases or a local recurrence at the site of the primary tumor. These are important predictors of overall survival since they determine whether local hepatic recurrences can still be treated [5]. PET-CT is currently the modality of choice to rule out extra-hepatic disease [33], but if PET-MRI proves to be effective in local follow-up future studies should focus on extrahepatic follow-up and the ability and feasibility of PET-MRI to detect metastatic lesions throughout the body. In addition, randomised controlled trials should be performed to compare PET-CT and PET-MRI, or when one of the techniques has proven to be significantly superior, an intense versus a less intense follow-up protocol after thermal ablation of CRLM and the effect on overall survival.

PET-MRI is currently only available in highly specialized hospitals. Before general implementation can be pursued, the potential and limitations of this modality need to be thoroughly investigated. In the current climate, associated costs also play an important role in the implementation of diagnostic and treatment methods in daily patient care. We are aware of only one study to date that has addressed the cost-effectiveness of PET-CT after RFA of hepatic malignancies [10]. These authors showed that PET-CT is more cost-effective than independent use of CT or MRI. However, as no study has yet addressed the cost-effectiveness of using PET-MRI, long-term cost-effectiveness, the results of future treatment and the yield of different scan-moments all need to be assessed.

This study will contribute to the search for an optimal follow-up protocol for patients treated with RFA or MWA for CRLM. As minimally invasive procedures become increasingly important in the treatment of the oncologic patient, adequate staging of disease is essential for optimal treatment results. This study may contribute to improved staging and consequently to the effective treatment of this ever-expanding patient group.

## Abbreviations

18-FDG: Fluorine-18 deoxyglucose; ceCT: Contrast enhanced computed tomography; CRLM: Colorectal liver metastases; DW-MRI: Diffusion-weighed magnetic resonance imaging; eGFR: Estimated glomerulal filtration rate; GRE: Gradient echo; keV: Kilo-electronvolt; LTP: Local tumor progression; mAs: Mili amperes; MRI: Magnetic resonance imaging; MWA: Microwave ablation; PET: Positon emmission tomography; RFA: Radiofrequency ablation; TR/TE: Repetition time/echo time; TSE: Turbo spin echo.

## Competing interests

The authors declare that they have no competing interests and there is no funding to be reported. Our study protocol has not undergone peer-review by a funding body.

## Authors’ contributions

KN: study conception and design, collection data on the background, drafting and revising the article. HJS: study conception and design, drafting and revising the article. ICP: design of the study, design of the manner of evaluation, expertise on MRI sequences, revising the article. AvT: Assistance in conception of the logistics, assistance in the practical solutions regarding PET-MRI. Revising the manuscript. JvW: Substantial assistance in conception of the MRI protocol, revising the manuscript. DEO: Substantial assistance in conception of the PET protocol, revising the manuscript. MRM: Substantial assistance in conception of the 4-phase CT protocol, revising the manuscript. GK: substantial assistance in conception of the follow-up system, corrected the manuscript. OSH: Assistance in the study design, statistical analysis and epidemiology. Advised on PET protocols. Revising the manuscript. WHS: Substantial contribution in the study design, revised and corrected different versions of the manuscript. CS: Implementation of this project in Gelderse Vallei Ede, advise on practical aspects of the study, revising the article. SM: Critical notes on study design and methods on data analyses, corrected and discussed all versions of the manuscript. EFIC: Supervising the information on nuclear imaging, co-initiator of the project, assisted in writing the manuscript and approved final version. MPT: Co-initiator of the study, supervisor of the whole study, corrected and discussed all versions of the manuscript. All authors have read and approved the final version submitted and agree to be accountable for all aspects of the work in ensuring that questions related to the accuracy or integrity of any part of the work are appropriately investigated and resolved.

## Pre-publication history

The pre-publication history for this paper can be accessed here:

http://www.biomedcentral.com/1471-2342/14/27/prepub
